# Discovering the indigenous microbial communities associated with the natural fermentation of sap from the cider gum *Eucalyptus gunnii*

**DOI:** 10.1038/s41598-020-71663-x

**Published:** 2020-09-07

**Authors:** Cristian Varela, Joanna Sundstrom, Kathleen Cuijvers, Vladimir Jiranek, Anthony Borneman

**Affiliations:** 1 The Australian Wine Research Institute, Glen Osmond, PO Box 197, Adelaide, SA 5064 Australia; 2 Department of Wine and Food Science, University of Adelaide, Glen Osmond, Adelaide, SA 5064 Australia

**Keywords:** Microbial ecology, Microbial communities

## Abstract

Over the course of human history and in most societies, fermented beverages have had a unique economic and cultural importance. Before the arrival of the first Europeans in Australia, Aboriginal people reportedly produced several fermented drinks including *mangaitch* from flowering cones of Banksia and *way-a-linah* from Eucalyptus tree sap. In the case of more familiar fermented beverages, numerous microorganisms, including fungi, yeast and bacteria, present on the surface of fruits and grains are responsible for the conversion of the sugars in these materials into ethanol. Here we describe native microbial communities associated with the spontaneous fermentation of sap from the cider gum *Eucalyptus gunnii*, a Eucalyptus tree native to the remote Central Plateau of Tasmania. Amplicon-based phylotyping showed numerous microbial species in cider gum samples, with fungal species differing greatly to those associated with winemaking. Phylotyping also revealed several fungal sequences which do not match known fungal genomes suggesting novel yeast species. These findings highlight the vast microbial diversity associated with the Australian *Eucalyptus gunnii* and the native alcoholic beverage *way-a-linah*.

## Introduction

Since the establishment of human settlement, alcoholic beverages have played a unique role in most societies, due to their economic and cultural importance^1^. Archaeological evidence suggests that the oldest fermented beverage was produced in Henan province, Northern China between 7000 and 6650 BCE and which was made of grapes, hawthorn berries, honey, and rice^2^. Evidence has also dated wine production in Iran as early as 6000 BCE and in Egypt to around 3000 BCE^3,4^. Since that time, it is believed that the use of fermentation to produce alcoholic beverages expanded from Mesopotamia throughout the world^1^. It is very likely that the consumption of alcoholic foods and/or beverages precedes modern humans, with frugivorous species, including many primates and hominoid lineages, eating and developing a taste for fermented fruits^5,6^. Indeed, evidence indicates that the ancestor of humans, chimpanzees, and gorillas adapted to metabolise ethanol long before human-directed fermentation^7^.

Early communities produced fermented beverages from different substrates, including honey to make mead or honey wine, which was produced in Asia around 1700–1100 BCE; grapes and barley to make wine and beer, respectively, in the Middle east, Rome and China^8^. In South America grains or fruits were used to produce *chicha*, while in North America, *octli* (now known as *pulque*) was made from agave, a type of cactus^8^. In Eastern Europe and central Asia, mare’s milk was used to produce a lactic-alcoholic beverage called *koumiss*^9^. In Australia before the arrival of the first Europeans, Aboriginal people produced several fermented drinks including *mangaitch* from flowering cones of Banksia in Western Australia, *way-a-linah* from Eucalyptus tree sap in Tasmania and *kambuda* from crushed nuts of the palm-like Pandanus tree in the Northern Territory^10^. Torres Strait Islanders produced *tuba* from the fructifying buds of coconut palms, a knowledge gained from Southeast Asian populations and which spread throughout the Pacific^11^.

In the case of *mangaitch*, Aboriginal people would dig a trench near a swamp and then line the excavation with a boat-shaped container made of tea-tree bark^10^. This container was then filled with water and Banksia cones and left to soak to obtain a sugar-rich solution, which was then left to ferment for several days. The drink known as *way-a-linah* was made by Tasmanian *Palawa* people from the sap of *Eucalyptus gunnii*. Stone tools were used to bore a hole in the trunk of the tree and to make a larger depression at the base to collect the flowing sap, which was then left to ferment after covering the collecting hole with a flat stone^10^. Without human intervention, sap also flows from naturally occurring holes in the trunk and accumulates at the base of the tree. For *kambuda*, reported from the Borroloola region, only ripe Pandanus nuts were used, which were fire roasted and then crushed with a stone, with the resulting pulp soaked in water for two days in a bark dish before fermentation^10^. *Tuba* was made from the syrup that seeps from a cut made in the unopened fructifying bud of *Cocos nucifera*, which was collected and left to ferment for several days^11^.

The alcohol present in fermented beverages is the result of yeast metabolism which converts the sugars present in fruits, grains, milk, honey and other carbon sources, into ethanol^12^. This process was named fermentation, from the Latin word *fervere*, which means ‘to boil’, probably due to the bubbles of CO_2_ produced by yeast^8^. Undoubtedly, the first fermented beverages were the result of spontaneous fermentation by microorganisms (fungi, yeast and bacteria) present on the surface of fruits and grains, inside fermentation pots or inadvertently introduced by human action^12^.

Spontaneous fermentation, is a complex microbial process involving the action of different species, which not only generate ethanol but also produce numerous secondary metabolites that shape the aroma and flavour of fermented beverages^12,13^. Although numerous indigenous fermented beverages have been produced worldwide^14^, only a few have been characterised from a microbiological perspective^15^. These include: *tej*, an Ethiopian honey wine^16^; *chicha*, a maize fermented drink from Peru^17^ or a rice-based fermented beverage from Brazil^18^; *pulque*, a Mexican fermented beverage produced from the sap of agave plants^19,20^; and *makgeolli*, a Korean traditional alcoholic beverage made from rice^21^, among others. Nowadays, spontaneous fermentation is used in the commercial production of several fermented beverages^13,22–24^. However, the microbial communities associated with the production of certain beer styles (e.g. Belgian lambic beer and American Coolship Ales) and related to wine fermentation are the most thoroughly characterised^23,25–28^.

*E. gunnii*, also known as cider gum, is a tree species endemic to Tasmania which is mainly found in cold, waterlogged habitats, such as lake edges, or poorly drained valley flats^29^. Factors such as climate change, possum and invertebrate activity, and stock grazing have been linked to a severe *E. gunnii* decline^30^. Particularly in the Central Plateau region of Tasmania, increased temperatures and a reduction in summer rainfall are contributing to a significant decrease in the availability of suitable micro‐sites for the successful regeneration of *E. gunnii*^31^. In this work, *E. gunnii* trees from three geographical locations in the Tasmanian Central Plateau in Australia, were sampled in two different dates to collect sap, bark and soil samples. Thus, we report for the first time the native microbial communities associated with *E. gunnii* and reveal major differences with the microbial composition associated with the production of other fermented beverages.

## Results

**Figure 1:**
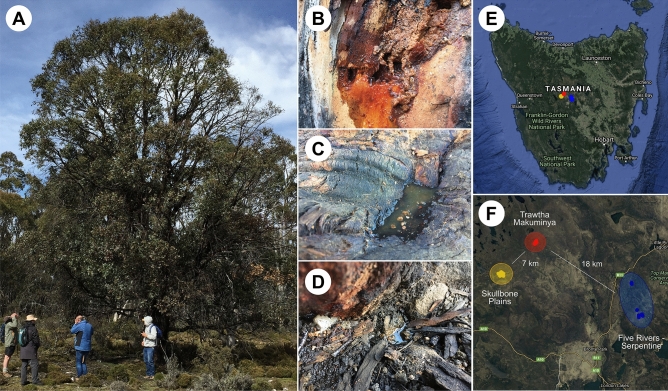
The Tasmanian cider gum *Eucalyptus gunnii* (**A**). Sap flows from naturally occurring holes in the trunk (**B**) and accumulates on the tree roots (**C**) and or on the soil (**D**). Bark, sap and soil samples were collected on the Tasmanian Central Plateau (**E**) from three different locations, Trawtha Makuminya (red), Skullbone Plains (yellow) and Five Rivers—Serpentine (blue) (**F**). Maps were created with ggmap version 3.0.0 in R version 3.6.2.

The Tasmanian *Eucalyptus gunnii* produces a sweet sap that often flows from naturally occurring holes in the trunk and accumulates at the tree roots and/or on the soil, where it ferments spontaneously (Fig. 1). To evaluate the microbial communities responsible for the fermentation of the cider gum sap we sampled 33 trees from three geographical locations in the Tasmanian Central Plateau in Australia, Trawtha Makuminya, Skullbone Plains and Five Rivers – Serpentine in November 2016 and January 2017 (Fig. 1). In total, 84 different samples, 45 from sap, 29 from bark and 10 from soil, were collected.

### Chemical composition of *Eucalyptus gunnii* sap

Sap samples were first analysed by HPLC to evaluate the presence of sugars, organic acids, glycerol and ethanol. A major peak, present in several samples, did not match any of the standards routinely used to quantify fermentation sugars and organic acids. GC/MS analysis on derivatised samples enabled the identification of this unknown compound as maltose. After identification, maltose was included with HPLC standards to obtain the basic chemical composition of *E. gunnii* sap (Table 1). Three sugars, glucose, fructose and maltose, were present in sap samples with maxima over 150 g/L. Several organic acids were also found with acetic and gluconic acids exceeding 30 g/L in some cases. Ethanol ranged from 0–6.1% (v/v).

**Table 1:** Basic chemical composition of sap collected from *Eucalyptus gunnii*.

Compound	Mean	Range
Fructose (g/L)	57.8	0.0–221.5
Glucose (g/L)	74.9	0.0–304.6
Maltose (g/L)	42.6	0.0–154.2
Acetic acid (g/L)	7.7	0.0–38.8
Ethanol (% v/v)	1.2	0.0–6.1
Gluconic acid (g/L)	15.2	0.1–68.6
Glycerol (g/L)	3.1	0.0–10.8
Pyruvic acid (g/L)	0.3	0.0–1.5
Succinic acid (g/L)	0.2	0.0–1.2

### Bacterial populations associated with *E. gunnii*

**Figure 2:**
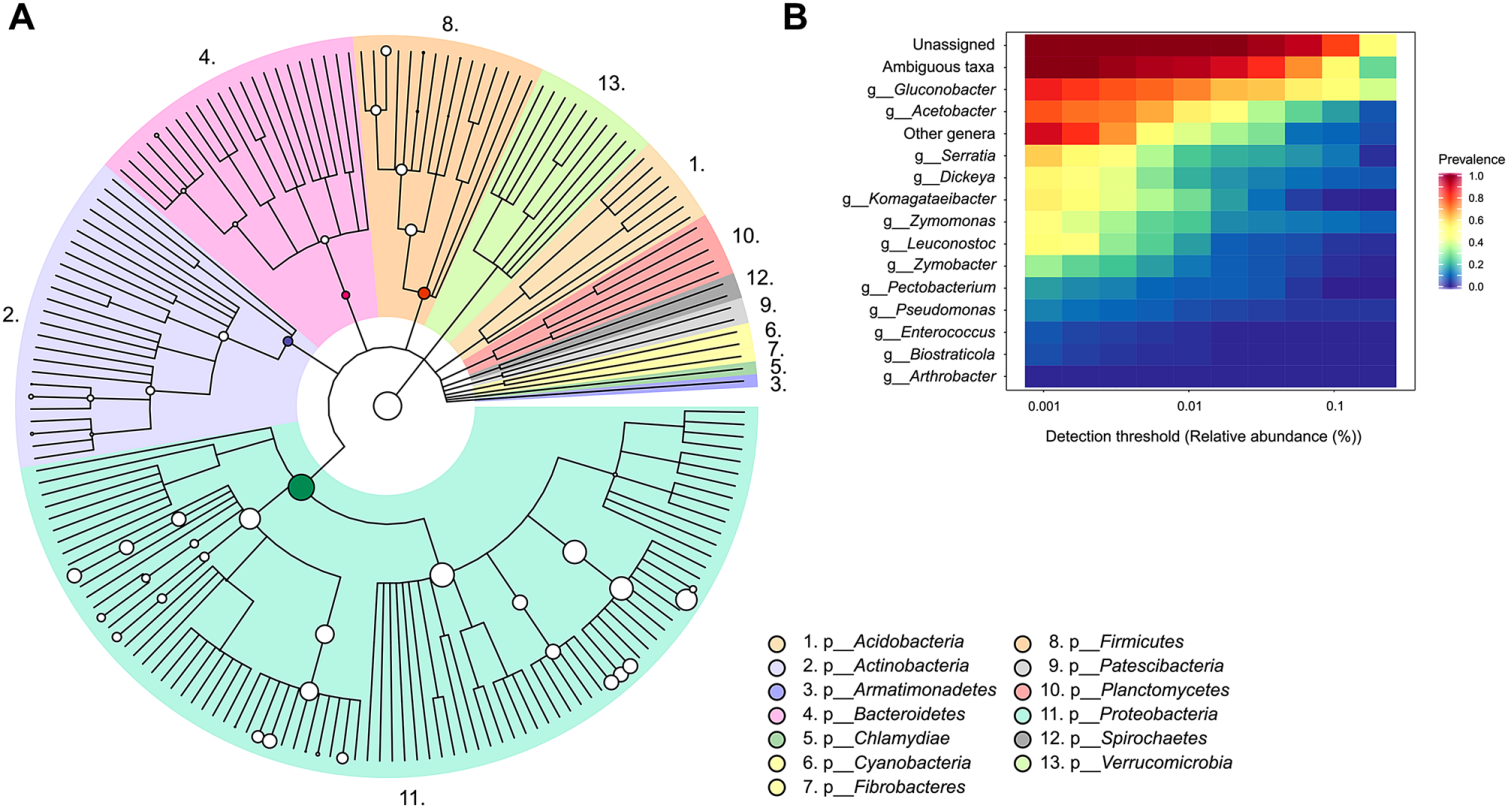
Bacterial communities associated with *Eucalyptus gunnii*. (**A**) The cladogram shows different taxonomy levels in concentric rings. From the centre, kingdom, phylum, class, order, family and genus are shown. All identified bacterial phyla have been coloured, with taxa abundance proportional to circle size. (**B**) Most prevalent bacterial genera across samples are shown at different detection thresholds.

Sap, bark and soil samples were assessed by 16S phylotyping to identify the bacterial communities associated with *E. gunnii.* Thirteen different phyla were found across the samples, regardless of geographical location, sample type or sampling date (Table S1), with *Proteobacteria* (65%), *Firmicutes* (3%), *Actinobacteria* (2%) and *Bacteroidetes* (1%) being the most abundant, while 28% of all OTUs could not be assigned to any phylum (Table S2). Small but significant differences were found between phyla when comparing different ecological niches, with soil samples containing higher proportions of *Fibrobacteres*, *Patescibacteria* and *Spirochaetes* (Table S2). A total of 26 classes, 61 orders, 93 families and 114 unique identified genera were found across all samples (Table S1), with a great diversity of genera for the phylum *Proteobacteria* (Fig. 2A). The phyla *Actinobacteria*, *Bacteroidetes* and *Firmicutes* also showed a considerable diversity in bacterial genera. Prevalent genera across all samples were *Gluconobacter*, *Acetobacter*, *Serratia*, *Dickeya*, *Komagataeibacter*, *Zymomonas* and *Leuconostoc* (Fig. 2B), with *Gluconobacter*, *Acetobacter* and *Zymomonas* also being the most abundant (Figure S1). Although small differences were observed when comparing the relative abundance of the same genus by geographical location, ecological niche or sampling date (Figure S2), most differences were not statistically significant. Only some samples from Five Rivers—Serpentine showed significant differences, with the genus *Enterococcus* exhibiting higher relative abundance in soil samples, whereas the genus *Gluconobacter* showed lower abundance in sap samples. Similarly, the genus *Komagataeibacter* showed a higher relative abundance in samples from November 2016 than from January 2017.

Significant differences were also found for diversity indices when samples were analysed by geographical location, ecological niche and sample date (Table 2). These indices included: richness, which indicates the number of species in an ecological community; diversity estimated with the Shannon index, which quantifies the uncertainty in the species identity of a random individual from the dataset; dominance, which indicates the abundance of a particular taxon over others; and divergence, which is calculated as the average dissimilarity of each sample from the group mean ^32^. Trawtha Makuminya showed higher diversity than other locations and lower dominance than Five Rivers—Serpentine. Soil samples had higher richness than sap samples, whereas bark samples showed lower divergence than sap and soil samples. Increased divergence was also found in samples obtained in January 2017.

**Table 2:** Diversity indices for bacterial and yeast communities associated with the cider gum, *Eucalyptus gunnii.*

	Location			Niche			Date	
	Five Rivers—Serpentine	Skullbone Plains	Trawtha Makuminya	Bark	Sap	Soil	Nov 2016	Jan 2017
**Bacterial communities**								
Richness	243.43^a^	182.27^a^	280.78^a^	248.17^ab^	243.31^b^	349.70^a^	271.85^a^	244.11^a^
Diversity^1^	2.30^b^	2.22^b^	2.95^a^	2.44^a^	2.72^a^	3.16^a^	2.77^a^	2.59^a^
Dominance	0.45^a^	0.47^ab^	0.33^b^	0.43^a^	0.36^a^	0.33^a^	0.35^a^	0.41^a^
Divergence	0.70^a^	0.69^a^	0.70^a^	0.65^a^	0.73^b^	0.71^ab^	0.67^a^	0.75^b^
**Fungal communities**								
Richness	206.48^a^	178.27^a^	184.58^a^	191.45^a^	207.98^a^	102.80^b^	190.32^a^	189.21^a^
Diversity^1^	2.44^a^	2.39^a^	2.31^a^	2.40^a^	2.32^a^	1.92^a^	2.25^a^	2.35^a^
Dominance	0.39^a^	0.32^a^	0.36^a^	0.34^a^	0.37^a^	0.39^a^	0.38^a^	0.35^a^
Divergence	0.78^a^	0.64^b^	0.77^a^	0.68^a^	0.81^b^	0.65^a^	0.76^a^	0.78^a^

^1^Diversity estimated by the Shannon index.

Different letters indicate significative differences (p < 0.05) for geographical location, ecological niche or sampling date.

### Fungal communities associated with *E. gunnii*

Sap, bark and soil samples were also analysed by ITS phylotyping to identify the fungal communities associated with *E. gunnii*. Only four fungal phyla were found, comprising 14 classes, 38 orders, 75 families, 136 genera and 204 unique identified species (Table S3). The most abundant classes included *Saccharomycetes*, *Dothideomycetes*, *Tremellomycetes*, *Leotiomycetes* and *Sordariomycetes*, while nearly 50% of all OTUs could not be assigned to any class (Table S4). Significative differences in abundance for different classes were found depending on geographical location, ecological niche or sampling date. In particular, *Dothideomycetes* were more abundant in Five Rivers—Serpentine, while *Eurotiomycetes* were more abundant in samples from Skullbone Plains. Sap samples showed high abundance in *Agaricomycetes*, *Cystobasidiomycetes*, *Dothideomycetes*, *Eurotiomycetes* and *Sordariomycetes.* Only *Agaricomycetes* showed a significant difference according to sampling date with higher abundance in samples from 2017 (Table S4).

**Figure 3:**
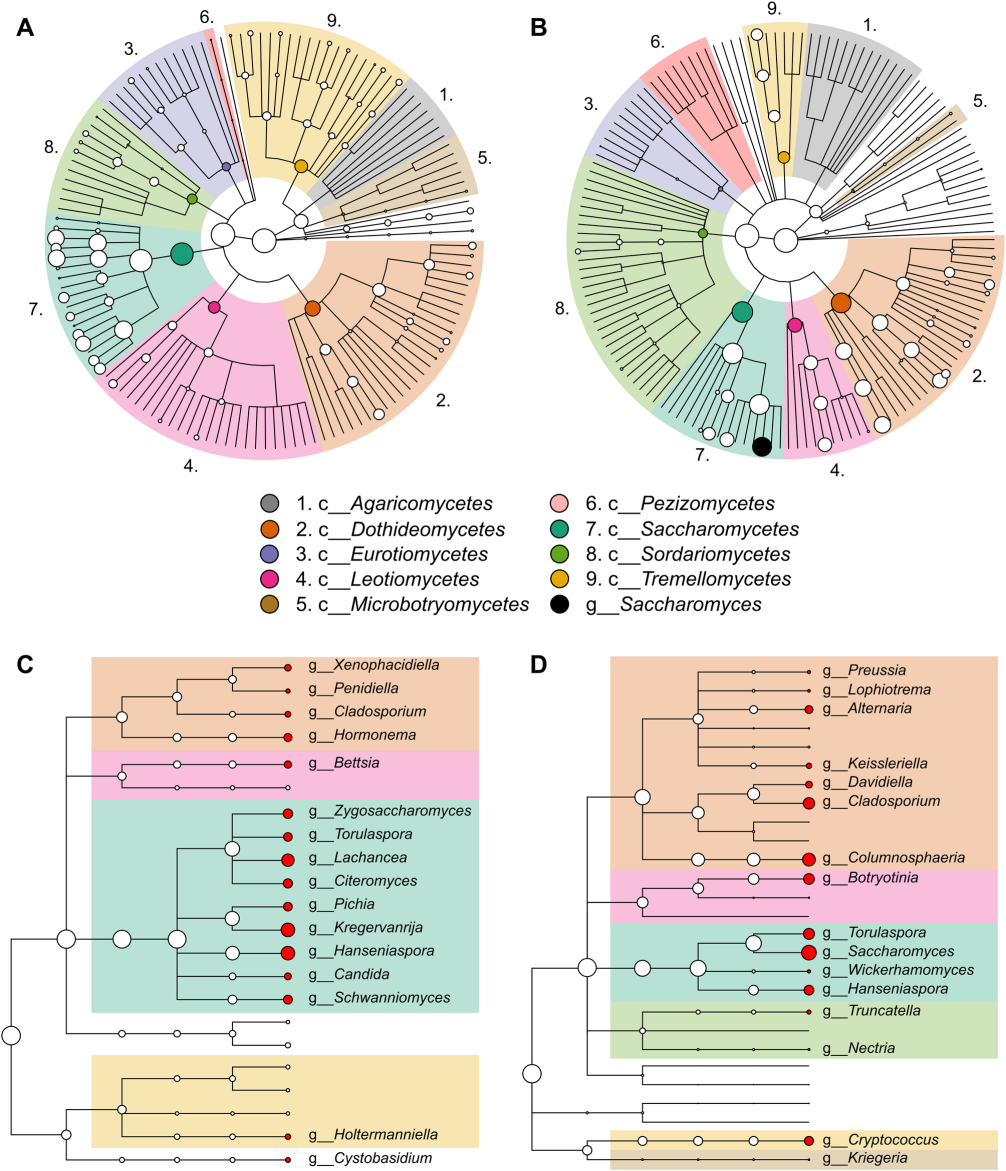
Comparison between yeast communities associated with *Eucalyptus gunnii* (**A**) and with *Vitis vinifera* (**B**). Cladograms show different taxonomy levels in concentric rings. From the centre, kingdom, phylum, class, order, family and genus. The main 8 classes have been coloured, whereas the genus *Saccharomyces* is indicated in black. Taxa abundance is proportional to circle size. The 15 most abundant yeast genera associated with *E. gunnii* (**C**) and with *V. vinifera* (**D**). *V. vinifera* data from Morrison-Whittle and Goddard^33,34^.

A similar diversity in the number of unique genera was observed for the classes *Leotiomycetes*, *Dothideomycetes*, *Saccharomycetes* and *Tremellomycetes* (Fig. 3A). The most prevalent genera were *Kregervanrija*, *Hanseniaspora*, *Lachancea*, *Zygosaccharomyces*, *Candida* and *Pichia* (Figure S4A). Diversity indices showed some significant differences when samples were analysed by geographical location and ecological niche (Table 2). Five Rivers—Serpentine showed higher divergence than Skullbone Plains, while sap samples showed increased divergence compared to bark and soil samples. No significant differences were found for sampling date.

### Relative abundances in fungal communities

**Figure 4:**
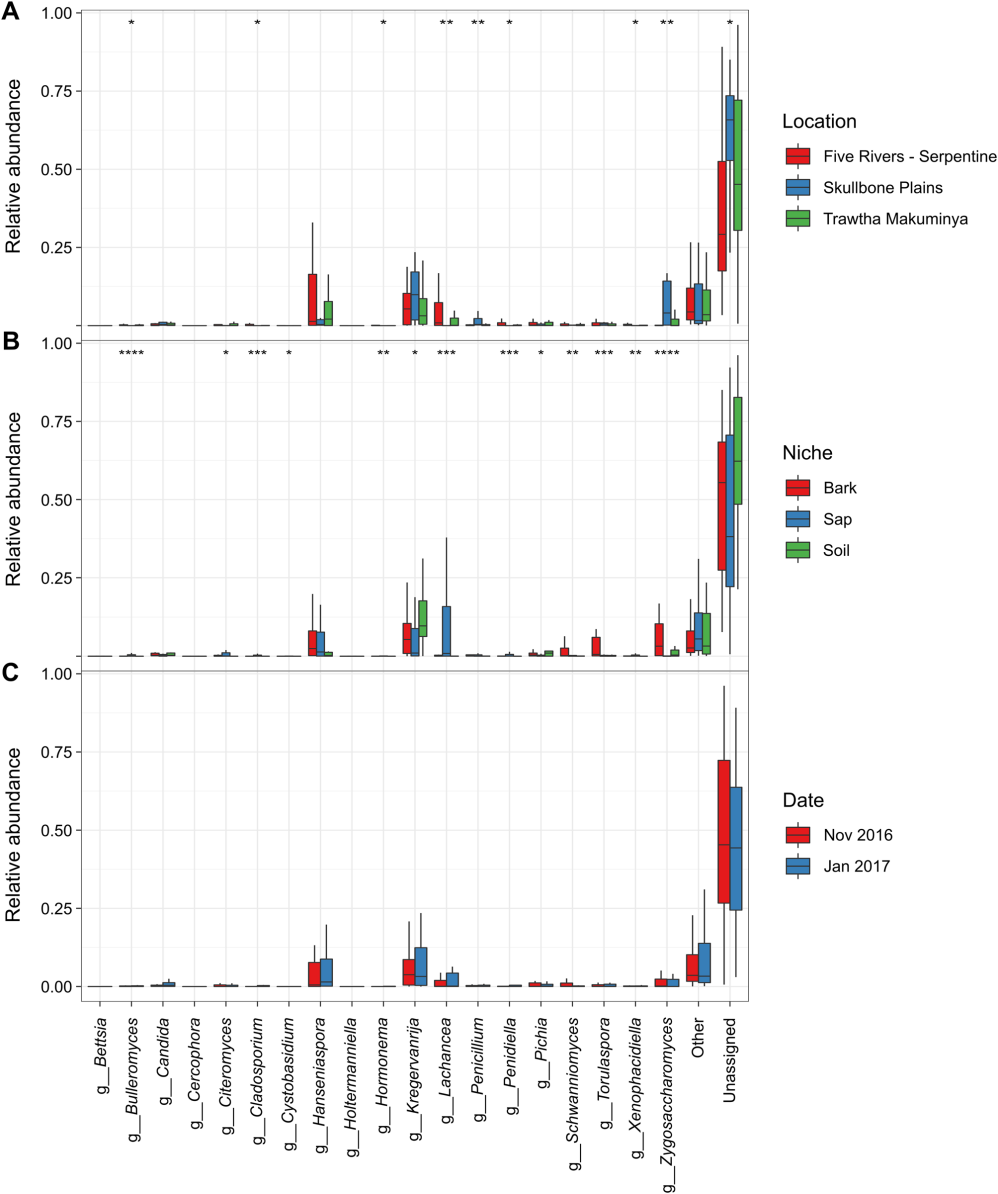
Boxplots showing relative abundance for the 15 most abundant fungal genera associated with *Eucalyptus gunnii* based on geographical location (**A**), ecological niche (**B**) and sampling date (**C**). Stars indicate statistically significative differences according to the Kruskal–Wallis test (*p < 0.05; **p < 0.01; ***p < 0.001).

Significative differences in relative abundance for the most abundant fungal genera were only found for geographical location and ecological niche (Fig. 4). The genera *Bulleromyces*, *Cladosporium*, *Hormonema*, *Lachancea*, *Penicillium*, *Penidiella*, *Xenophacidiella* and *Zygosaccharomyces* showed different abundances depending on geographical location, while *Bulleromyces*, *Citeromyces*, *Cladosporium*, *Cystobasidium*, *Hormonema*, *Kregervanrija*, *Lachancea*, *Penicillium*, *Penidiella*, *Pichia*, *Schwanniomyces*, *Torulaspora*, *Xenophacidiella* and *Zygosaccharomyces* differed according to ecological niche. Interestingly, the genus *Lachancea* had the lowest abundance in samples from Skullbone Plains and the highest in sap samples. On the other hand, the genus *Zygosaccharomyces* showed the highest abundance in samples from Skullbone Plains and in bark samples (Fig. 4, Figure S3). The most abundant fungal species were *Kregervanrija delftensis*, *Hanseniaspora valbyensis*, *Lachancea quebecensis*, *Citeromyces hawaiiensis*, *Schwanniomyces pseudopolymorphus*, *Lachancea cidri* and *Torulaspora globose* (Table S5). When samples were grouped according to geographical location some significative differences were found for sampling date (Figure S5). In Five Rivers – Serpentine, the genera *Mortierella* and *Torulaspora* differed in relative abundance according to sampling date, while in Trawtha Makuminya the genera *Cladosporium*, *Hanseniaspora*, *Lachancea*, *Metschnikowia* and *Penidiella* showed different relative abundance.

**Figure 5:**
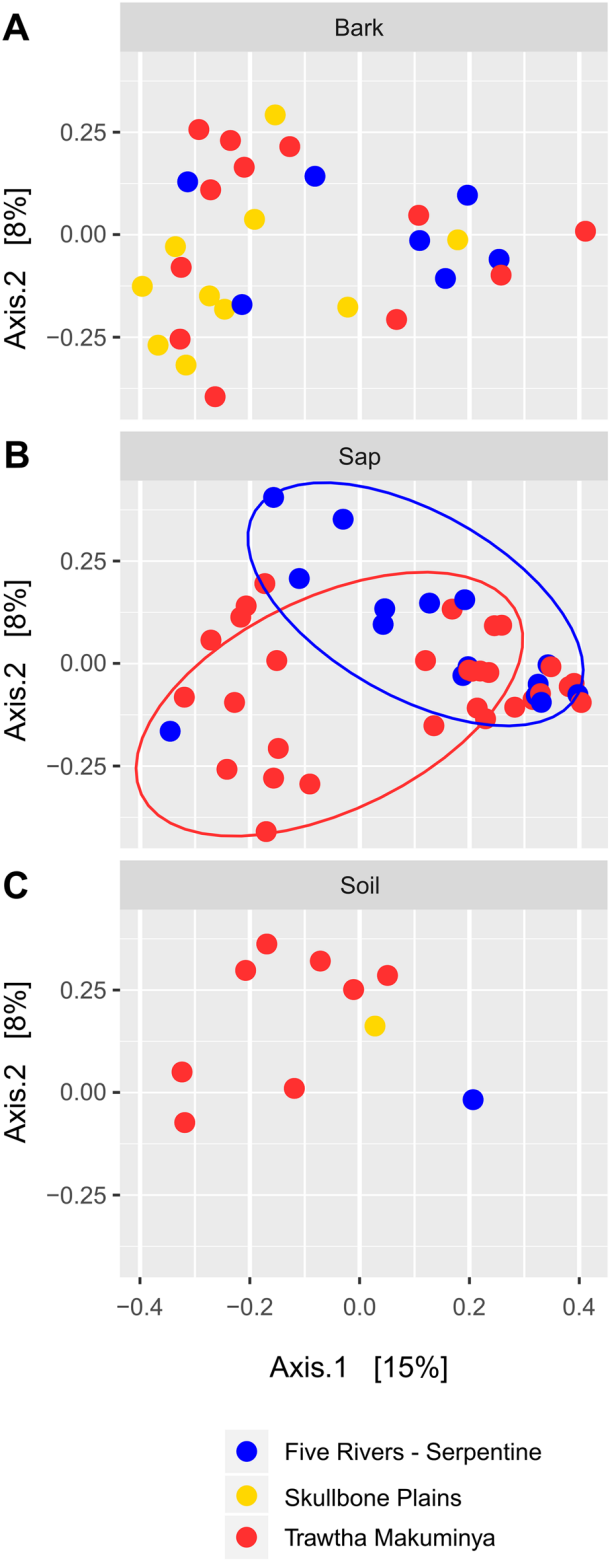
Principal Coordinates Analysis (PCoA) for the top 100 fungal OTUs in Five Rivers—Serpentine (blue), Skullbone Plains (yellow) and Trawtha Makuminya (red) for bark (**A**), sap (**B**) and soil (**C**) samples.

Permutational multivariate analysis of variance (PERMANOVA) showed that only ecological niche significantly affected the composition of fungal communities (Table 3). Geographical location, sampling data or the interaction between niche and location did not affect the diversity or composition of fungal populations. Principal Coordinates Analysis (PCoA) enabled to visualise clustering of sap samples according to geographical location for Trawtha Makuminya and Five Rivers—Serpentine (Fig. 5). No clustering was observed for bark or soil samples.

**Table 3:** Permutational multivariate analysis of variance (PERMANOVA) of Bray distances for fungal communities (999 permutations).

	Df	SS	MS	F model	R^2^	P value	Significance
Niche	2	1.959	0.980	2.582	0.059	< 0.001	***
Location	2	0.914	0.457	1.205	0.028	0.164	
Niche x Location	3	1.418	0.473	1.246	0.043	0.062	
Residuals	76	28.836	0.379		0.870		

*Df* degrees of freedom, *SS* sequential sum of squares, *MS* mean square.

### Fungal communities found in cider gum differed to those associated to winemaking

To compare the fungal communities associated with *E. gunnii*, which we obtained from soil, bark and sap, to those found in winemaking we used the data reported by Morrison-Whittle and Goddard^33,34^, which described fungal populations found in vineyard soil, on vine bark and grape berries, and during wine fermentation. Although the same main fungal classes were found for both cider gum and winemaking, genus diversity and/or relative abundance differed (Fig. 3A,B). *Eurotiomycetes*, *Leotiomycetes*, *Microbotryomycetes* and *Tremellomycetes* showed greater genus diversity in populations associated to cider gum, whereas *Agaricomycetes*, *Pezizomycetes* and *Sordariomycetes* had more diversity in communities associated to winemaking. Although genus diversity was similar for *Dothideomycetes* and *Saccharomycetes*, genus relative abundance was very different. The most abundant genera for *Dothideomycetes* associated with *E. gunnii* included: *Hormonema*, *Xenophacidiella*, *Penidiella* and *Cladosporium*, while *Columnosphaeria*, *Cladosporium*, *Alternaria*, *Davidiella* and *Keissleriella* were the most abundant in winemaking (Fig. 3C,D). For *Saccharomycetes*, the most abundant genera in cider gum samples were *Hanseniaspora*, *Kregervanrija*, *Lachancea*, *Zygosaccharomyces*, *Citeromyces*, *Schwanniomyces*, *Torulaspora*, *Pichia* and *Candida*, whereas in winemaking samples *Saccharomyces*, *Torulaspora*, *Hanseniaspora* and *Wickerhamomyces* were the most abundant.

Differences between fungal communities associated with cider gum and with grape growing and winemaking also included the most prevalent genera (Figure S4). Only three of the 16 most prevalent genera, *Hanseniaspora*, *Torulaspora* and *Cladosporium*, were common to both fungal communities. For communities associated with winemaking the most prevalent genera included *Cladosporium*, *Columnosphaeria*, *Alternaria*, *Saccharomyces* and *Davidiella*. Interestingly, *Saccharomyces*, which is the main genus responsible for alcoholic beverage fermentation including beer, cider and wine, was barely found in cider gum samples (Table S3). Species belonging to this genus, *S. cerevisiae* and *S. uvarum*, were only observed in some samples. While *S. cerevisiae* was found in samples from both Trawtha Makuminya and Five Rivers—Serpentine, *S. uvarum* was only found in the last location.

## Discussion

Numerous indigenous fermented beverages have been produced worldwide^10,11,14^. These beverages are the result of microbial activity, which generally involves the concerted action of numerous bacterial, fungal and yeast species. Here, we report the microbial communities associated with *way-a-linah*, a mildly alcoholic beverage made from the sap of the cider gum *E. gunnii* by Aboriginal people in Tasmania.

*E. gunnii*, which is endemic to Tasmania, shows exceptional cold tolerance for a eucalyptus tree (down to − 20 °C for brief periods) and is now commonly planted as an ornamental tree across the British Isles and some parts of Western Europe^35^. *E. gunnii* has been studied as a source of bioenergy^36^; phytochemicals, including vitamins and fatty acids, given the particularly high concentration of tocopherols compared to other plants^37^; and essential oils, due to its antioxidant, antimutagenic and antibacterial properties^38^. *E. gunnii* sap contained a high concentration of sugars, a trait which is likely associated to its cold tolerance. Indeed, a positive correlation between cold resistance and total soluble carbohydrate content has been reported in *E. gunnii* cell lines^39^. While sucrose was the main sugar found in the leaves^37^, the most abundant soluble sugars found in cells were glucose, fructose, sucrose, stachyose and raffinose^39^. Here, we found glucose, fructose and maltose to be the main sugars in *E. gunnii* sap. These sugars are also the main components of honey^14^, while sucrose, glucose and fructose are the most abundant sugars in maple syrup^40^.

Several studies have shown biogeographic differentiation between microbial communities with differences between environmental niches and geographical locations, particularly for vineyards and microbial populations associated with winemaking^26,33,41,42^. Differences among microbial communities have been found at large geographical scales (> 100 km)^26,33,34,41^, medium scales (> 35 km)^43^, and small scales (< 2 km)^44^, indicating that local environmental variables, such as soil type, humidity and nutrient availability among others, can shape microbial populations even within the same vineyard. Microbial communities described here showed significant differences according to geographical location, with sampling sites located within a 20 km radius, suggesting a similar effect of local environmental variables such as those observed for vineyards and winemaking^43,44^. Interestingly, differences in fungal and bacterial community compositions in vineyards have been described as much clearer in fungi than in bacteria, with leaf and berry fungal community dissimilarities between locations increasing with geographic distance, while bacterial community dissimilarity did not correlate with geographic distance^43^. Bacterial communities associated with *E. gunnii* showed only minor differences according to geographical location or ecological niche while fungal populations differed significantly even at genus level, indicating the important role of the environment on shaping fungal communities^44,45^.

Numerous environmental and anthropogenic factors have been shown to influence the composition of microbial communities found in soil or associated with plant material. These include season for communities in subtropical low mountain forest soils^46^ or vintage for grape vines^26,47^; physical characteristics of the soil, including carbon and nitrogen content^48^; vine vegetative cycle^42^; topological characteristics of the land^49^; application of chemical fungicides and fertilizers^47,50,51^; agricultural management practices in crop plantations and vineyards^52–54^; land-use change^55^; and erosion in farmlands^56^. Although none of these factors were investigated here, it is likely that several parameters, including climatic conditions, rainfall and soil nutrient availability, or combinations of these factors, shape the microbial populations associated with *E. gunnii*. For example, no differences were found for fungal genera according to sampling date when assessing all samples, however significant changes in composition were found when individual geographical locations were compared. This suggests that for *E. gunnii*, climatic conditions (spring *vs* summer) only influence fungal communities when combined with other factors.

Considering multiple grape varieties and numerous geographical locations, the main bacterial phyla found associated with vineyards and winemaking are *Proteobacteria*, *Firmicutes*, *Actinobacteria* and *Bacteroidetes*^26,42^. The same four phyla were also found as the most abundant in cider gum samples. While the genera *Klebsiella* and *Pseudomonas* from the phylum *Proteobacteria* are the most abundant in grape must^42^, the main bacterial genera found in *pulque* fermentation include *Lactobacillus* and *Leuconostoc* from the *Firmicutes* phylum, and the *Proteobacteria*, *Zymomonas*, *Acetobacter* and *Gluconobacter*^19^. Different lactic acid bacteria (LAB) species have also been shown to dominate the fermentation of *tej* honey wine, including *Lactobacillus*, *Streptococcus*, *Leuconostoc*, and *Pediococcus*^16^; the fermentation of *makgeolli*, including *Enterococcus*, *Lactobacillus*, *Leuconostoc*, *Pediococcus*, *Weissella* and *Lactococcus*^21^; and the fermentation of *chicha*, including *Lactobacillus*, *Bacillus*, *Leuconostoc* and *Enterococcus*^18^. LAB species associated with *E. gunnii* represented < 3% of the total bacterial population, with *Leuconostoc* as the main genus, whereas the acetic acid bacteria (AAB) *Gluconobacter* and *Acetobacter*, were the most abundant bacterial genera. The higher proportion of AAB in cider gum samples highlights the distinctive bacterial communities associated with this Tasmanian drink compared to the bacterial populations involved in the production of other fermented beverages. This enrichment in AAB could be the result of the aerobic conditions in which *way-a-linah* fermentation occurs.

Although fungal communities associated with *E. gunnii* and those related to winemaking, including fungal populations found in vineyard soil, on vine bark and grape berries, and during wine fermentation, showed similar diversity, their taxonomic composition and relative abundances differed considerably, particularly for lower taxonomic levels. The most abundant fungal phyla, *Ascomycota* and *Basidiomycota*, as well as the most abundant fungal classes, *Saccharomycetes*, *Dothideomycetes* and *Leotiomycetes* were similar for communities associated with winemaking and *way-a-linah*^26,27,34,42^. However, the most abundant taxa for order, family and genus diverged for both communities. Interestingly, the main classes found in other Eucalyptus species differed from those associated with *E. gunnii,* with the most abundant classes found in leaves, twigs and trunks of *E. grandis* including *Dothideomycetes* and *Sordariomycetes* while *Saccharomycetes* represented only 0.05% of the population^57^. At family level, the main taxa associated with *E. grandis* included *Mycosphaerellaceae*, *Botryosphaeriaceae* and *Teratosphaeriaceae*^57^, whereas those related to *E. gunnii* were *Saccharomycetaceae*, *Pichiaceae* and *Saccharomycodaceae*. In cider gum *Teratosphaeriaceae* and *Mycosphaerellaceae* accounted for less than 3% of the total fungal population, whereas *Botryosphaeriaceae* was not found. This suggests that at higher taxa levels, fungal populations associated with *E. gunnii* resembled those found in vineyards and winemaking rather than those of other Eucalyptus species.

The genus *Saccharomyces*, which is the most abundant in winemaking^27,34,42^, was practically absent from cider gum samples, in which *S. cerevisiae* and *S. uvarum* were found at very low abundance (< 0.03%) and only in a small proportion of samples. *Saccharomyces* yeasts have a crucial competitive advantage during sugar fermentation due to their ability to grow fast, produce and consume ethanol and their tolerance for various environmental stresses^58^. The fact that *Saccharomyces* was not abundant, particularly in fermenting sap samples, suggest that this genus is hardly present in the locations sampled and/or that the conditions in cider gum sap, including the presence of other microorganisms, low alcohol concentration, high organic acid concentration, among others, may make the competition equal to other species.

We found numerous bacterial (close to 30%) and fungal (under 50%) OTUs, which did not match any entries in 16S rRNA and ITS databases, or matched ‘uncultured’ microorganisms providing no taxonomic information for those sequences. This has also been described in other environmental samples, in fact the highest abundances of bacterial OTUs belong to phylogenetically novel uncultured groups in seawater, freshwater, terrestrial subsurface, soil, hypersaline environments, marine sediment, hot springs, hydrothermal vents, nonhuman hosts, snow, and bioreactors^59^. Thus, not only most bacterial, but also most archaeal taxa remain uncultured and therefore uncharacterised^60^. Similarly, several studies have shown a significant abundance of unclassified or unassigned fungal OTUs^33,50,54,57^ suggesting the existence of many novel fungal taxa. Careful examination of culture conditions and meticulous formulation of laboratory culture media have enabled researchers to bring the uncultured into culture and provide essential information about novel species^61^. Ideally, applying this approach to cider gum samples would make possible the isolation and characterisation of novel bacterial and fungal species.

In conclusion, we have found numerous microbial species associated with the spontaneous fermentation of sap from *E. gunnii*. While bacterial communities showed small differences between geographical locations, ecological niches or sampling dates, fungal populations showed significant differences. Additionally, fungal communities differed greatly to those associated with winemaking. Phylotyping revealed several bacterial and fungal sequences, which did not match known microbial genomes suggesting potential novel microbial taxa. These findings highlight the vast microbial diversity associated with native Australian plants and beverages.

## Materials and methods

### Sampling

Bark, sap and soil samples were collected from the cider gum *Eucalyptus gunnii* on the Tasmanian Central Plateau in Australia. Samples were obtained from three different geographical locations, Trawtha Makuminya (41°59′55.6″S 146°22′58.8″E), Skullbone Plains (42°02′29.2″S 146°19′13.6″E) and Five Rivers—Serpentine (42°05′13.5″S 146°34′07.3″E) at two different dates in November 2016 and January 2017 (Fig. 1).

### Chemical analysis

Chemical analysis was performed only on sap samples. Preliminary analysis and compound identification were performed by gas-chromatography mass spectrometry (GC/MS) on an Agilent 7890 gas chromatograph equipped with Gerstel MPS2 multi-purpose sampler and coupled to an Agilent 5975C VL mass selective detector. Instrument control was performed with Agilent G1701A Revision E.02.00 ChemStation software. The gas chromatograph was fitted with a VF-5ms column (30 m × 250 μm × 0.25 μm) fitted with a 10 m guard column and helium (ultra high purity) was used as the carrier gas in constant flow mode at 1 mL/min. Samples were derivatised by adding an aliquot of 22 μL of *N*-methoxyamine hydrochloride (Sigma-Aldrich) in pyridine (Sigma-Aldrich) and incubating at 37 °C with agitation for 2 h. Following incubation, 4 μL of alkane standard mixture (Cat. No. 68281, Sigma-Aldrich) and 22 μL of *N*,*O*-Bis(trimethylsilyl)trifluoroacetamide and trimethylchlorosilane (BSTFA + TMCS, 99:1, Sigma-Aldrich) were added, and incubated at 37 °C with agitation for 60 min. Derivatised samples were incubated at room temperature for 1 h before injection. A volume of 1 μL of derivatized sample was injected into the inlet which was set at 250 °C in pulsed splitless mode. The oven program started at 35 °C for 2 min, then ramped at 10 °C/min to 300 °C for 16 min. Total run time was 46 min. The mass spectrometer quadrupole was set at 150 °C, the source was set at 250 °C while the transfer line was held at 280 °C. Positive ion electron impact spectra at 70 eV were recorded in scan mode with an m/z range of 50–600 and a solvent delay of 9.6 min. After confirming the identity of maltose by GC/MS, quantification of sugars, including glucose, fructose and maltose; organic acids, including gluconic, pyruvic, succinic and acetic acids; glycerol and ethanol was performed by high-performance liquid chromatography (HPLC) using a BioRad HPX87H column at 65 °C, H_2_SO_4_ 5 mM as mobile phase at 0.5 mL/min, as described previously^62^.

### DNA extraction

Bark samples were incubated at 4 °C for 24 h with 1 mL of PBS (NaCl 137 mM, KCl 2.7 mM, Na_2_HPO_4_ 10 mM, KH_2_PO_4_ 1.8 mM pH 7.4) before the liquid was used for DNA extraction. Total DNA was isolated from bark and sap samples using the DNeasy PowerFood Microbial kit (Qiagen, Hilden, Germany) as described by the manufacturer. DNA from soil samples was extracted using the DNeasy PowerSoil kit (Qiagen, Hilden, Germany) according to the manufacturer’s instructions. After isolation, DNA was quantified with the Qubit dsDNA HS kit assay (Thermo Fisher Scientific, Massachusetts, USA).

### Determination of microbial populations

Microbial populations were assessed using amplicon sequencing. Briefly, 1.0 ng of total DNA from each sample was subjected to a two-step PCR process that amplifies the V3-V4 region of the bacterial 16S rRNA^63^ or the ITS2 locus from the fungal ribosomal internal transcribed spacer (ITS) region^64^ while adding both custom in-line barcodes and sequences necessary for Illumina sequencing (including compatible Illumina dual-indexes) according to the process outlined previously^65^. Briefly, following sequencing, raw reads were quality and adaptor trimmed (Trimmomatic^66^; Cutadapt^67^), with paired-end reads overlapped^68^ to form a single contiguous synthetic read. Reads were then assigned to samples and timepoints using a combination of both the in-line and Illumina barcodes using custom Python scripts. Operational taxonomic units (OTUs) were clustered de novo using Swarm v2.0^69^ and taxonomies assigned using the “*assign*_*taxonomy.py*” functionality of QIIME^70^. Fungal taxonomy was assigned against the QIIME UNITE fungal ITS database (ver7 dynamic 20.11.2016), while bacterial taxonomy used the SILVA 16S database (version 132 QIIME release) as a reference. Sequences and abundances for all bacterial and fungal OTUs identified in this study are listed in Table S1 and Table S3, respectively.

### Data and statistical analyses

Data analysis and graphical representation were performed using the R packages phyloseq^71^, microbiome^32^, vegan^72^, microbiomeViz^73^, ggmap^74^, ggpubr^75^ and ggplot2^76^ in R version 3.6.2^77^. OTUs showing less than 0.001% abundance were not considered for analysis. Kruskal tests were used to evaluate statistical differences for relative abundances between species.

## Supplementary information

Supplementary information.

Supplementary Table S1.

Supplementary Table S3.
