# Supplementary material for: Discovering the indigenous microbial communities associated with the natural fermentation of sap from the cider gum Eucalyptus gunnii
Source: Sci Rep. 2020 Sep 7;10:14716. doi: 10.1038/s41598-020-71663-x (PMC7477236; doi:10.1038/s41598-020-71663-x)
Supplement: Supplementary file 1 — Supplementary information. [file 41598_2020_71663_MOESM1_ESM.pdf]

Discovering the indigenous microbial communities associated with the natural fermentation of sap from the cider gum *Eucalyptus gunnii*

Cristian Varela, Joanna Sundstrom, Kathleen Cuijvers, Vladimir Jiranek, Anthony Borneman

Table S1. Sequences and abundances for all bacterial OTUs.

Table S2. Relative abundance for all bacterial phyla (%).

		Location			Niche			Date	
Phylum	Overall	Five Rivers - Serpentine	Skullbone Plains	Trawtha Makuminya	Bark	Sap	Soil	Nov 2016	Jan 2017
p__*Acidobacteria*	0.087	0.032	0.091	0.111	0.116	0.066	0.094	0.079	0.094
p__*Actinobacteria*	1.952	3.677	0.133	1.558	1.651	2.458	0.545	0.187	3.634
p__*Armatimonadetes*	0.009	0.000	0.000	0.015	0.002	0.004	0.055	0.013	0.005
p__*Bacteroidetes*	1.348	0.098	3.347	1.484	1.721	1.000	1.831	1.466	1.236
p__*Chlamydiae*	0.004	0.000	0.000	0.007	0.000	0.008	0.000	0.000	0.009
p__*Cyanobacteria*	0.066	0.000	0.000	0.111	0.000	0.090	0.148	0.056	0.075
p__*Fibrobacteres*	0.003	0.000	0.000	0.005	0.000[b]	0.000[b]	0.026[a]	0.006	0.000
p__*Firmicutes*	3.199	2.181	1.620	4.014	1.512	3.380	7.276	3.208	3.190
p__*Patescibacteria*	0.023	0.003	0.051	0.026	0.025[b]	0.010[b]	0.075[a]	0.018	0.027
p__*Planctomycetes*	0.017	0.019	0.000	0.020	0.024	0.003	0.059	0.025	0.010
p__*Proteobacteria*	64.722	63.478	62.988	65.675	71.489	59.878	66.895	69.042	60.603
p__*Spirochaetes*	0.006	0.000	0.000	0.010	0.000[b]	0.000[b]	0.052[a]	0.013	0.000
p__*Verrucomicrobia*	0.285	0.000	0.021	0.474	0.013	0.440	0.374	0.570	0.013
Unassigned	28.279	30.513	31.749	26.489	23.448	32.662	22.570	25.317	31.104

Different letters indicate statistically significant differences ($p < 0.05$ according to Kruskal test)

Table S3. Sequences and abundances for all fungal OTUs.

Table S4. Relative abundance for all fungal classes (%).

Class	Overall	Location			Niche			Date	
		Five Rivers - Serpentine	Skullbone Plains	Trawtha Makuminya	Bark	Sap	Soil	Nov 2016	Jan 2017
c__*Agaricomycetes*	0.201	0.608	0.004	0.058	0.103[b]	0.288[a]	0.100[b]	0.062[b]	0.335[a]
c__*Cystobasidiomycetes*	0.547	1.083	0.012	0.418	0.008[b]	1.004[a]	0.054[b]	1.062	0.056
c__*Dothideomycetes*	7.279	15.940[a]	0.842[b]	4.712[b]	4.105[b]	9.509[a]	6.451[b]	3.711	10.682
c__*Eurotiomycetes*	0.971	0.702[b]	1.977[a]	0.874[b]	1.044[a]	1.095[a]	0.204[b]	0.703	1.227
c__*Leotiomycetes*	1.459	0.663	2.697	1.553	2.262	1.236	0.140	1.832	1.105
c__*Microbotryomycetes*	0.295	0.688	0.060	0.166	0.026	0.253	1.266	0.418	0.178
c__*Mortierellomycetes*	0.685	2.441	0.005	0.026	0.093[a]	1.219[a]	0.000[b]	0.032	1.308
c__*Mucoromycetes*	0.001	0.000[b]	0.010[a]	0.000[b]	0.004	0.000	0.000	0.000	0.002
c__*Orbiliomycetes*	0.064	0.002	0.006	0.106	0.184	0.001	0.002	0.129	0.002
c__*Pezizomycetes*	0.062	0.000	0.443	0.006	0.179	0.000	0.000	0.008	0.113
c__*Saccharomycetes*	38.177	37.748	31.997	39.735	42.216	38.148	26.599	38.840	37.545
c__*Sordariomycetes*	0.508	0.428[a]	0.158[b]	0.622[a]	0.124[b]	0.808[a]	0.273[b]	0.771	0.257
c__*Tremellomycetes*	2.487	3.322	0.864	2.461	0.789[b]	3.661[a]	2.132[a]	2.202	2.759
c__*Wallemiomycetes*	0.021	0.003	0.067	0.020	0.002[b]	0.015[b]	0.107[a]	0.010	0.033
Unassigned	47.240	36.373[b]	60.859[a]	49.243[b]	48.863	42.765	62.673	50.221	44.398

Different letters indicate statistically significant differences ($p < 0.05$ according to Kruskal test)

Table S5. Most abundant fungal species (%).

Species	Overall
s__*Kregervanrija delftensis*	9.308
s__*Hanseniaspora valbyensis*	6.684
s__*Lachancea quebecensis*	3.182
s__*Citeromyces hawaiiensis*	2.465
s__*Schwanniomyces pseudopolymorphus*	2.436
s__*Lachancea cidri*	2.204
s__*Torulaspora globosa*	2.082
s__*Pichia kluyveri*	1.865
s__*Hormonema macrosporum*	1.348
s__*Hanseniaspora thailandica*	1.285
s__*Candida salmanticensis*	1.193
s__*Penidiella carpentariae*	0.915
s__*Cladosporium delicatulum*	0.709
s__*Bettsia alvei*	0.395
s__*Holtermanniella takashimae*	0.146
s__*Cercophora vinosa*	0.109
Unassigned	47.240
Other[1]	11.770
Ambiguous taxa	4.666

[1] identified species with abundance lower than 0.1%

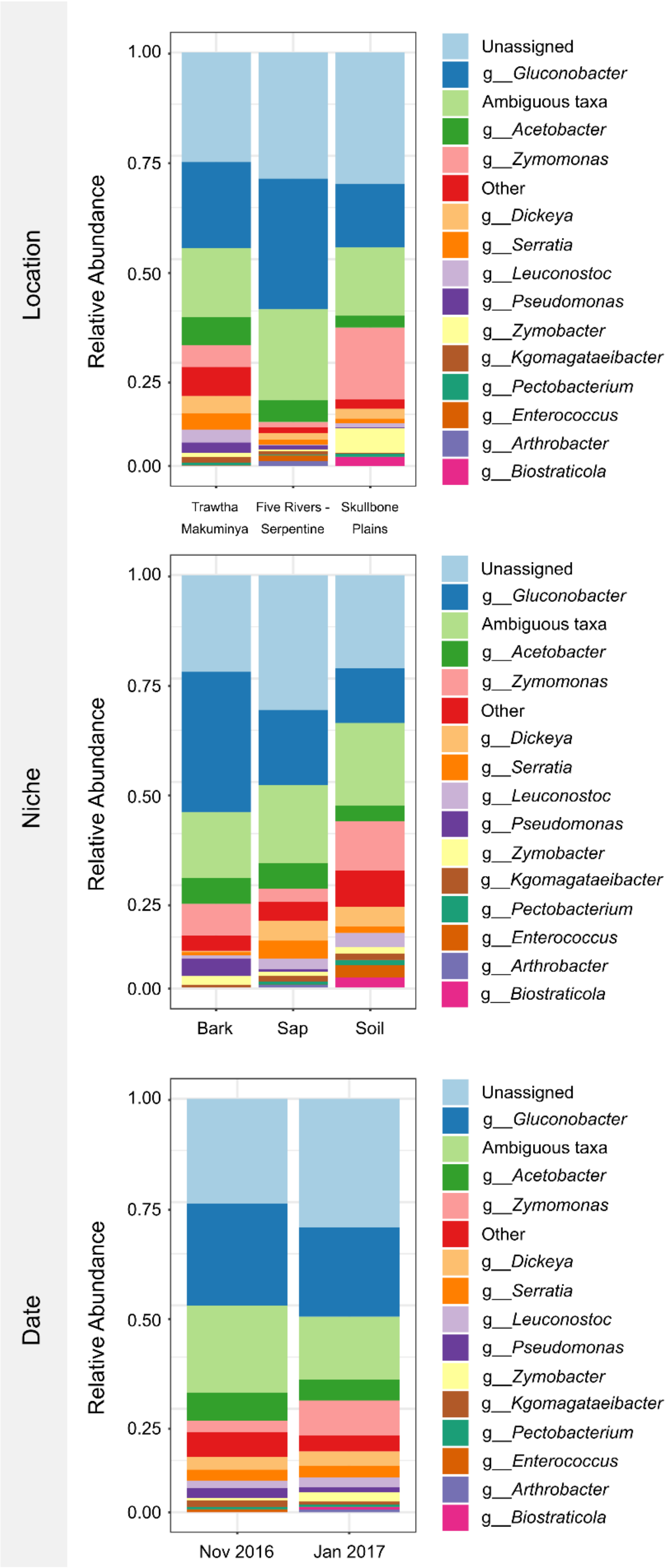

Figure S1. Relative abundance for the most abundant bacterial genera associated with *Eucalyptus gunnii* according to geographical location, ecological niche or sampling date.

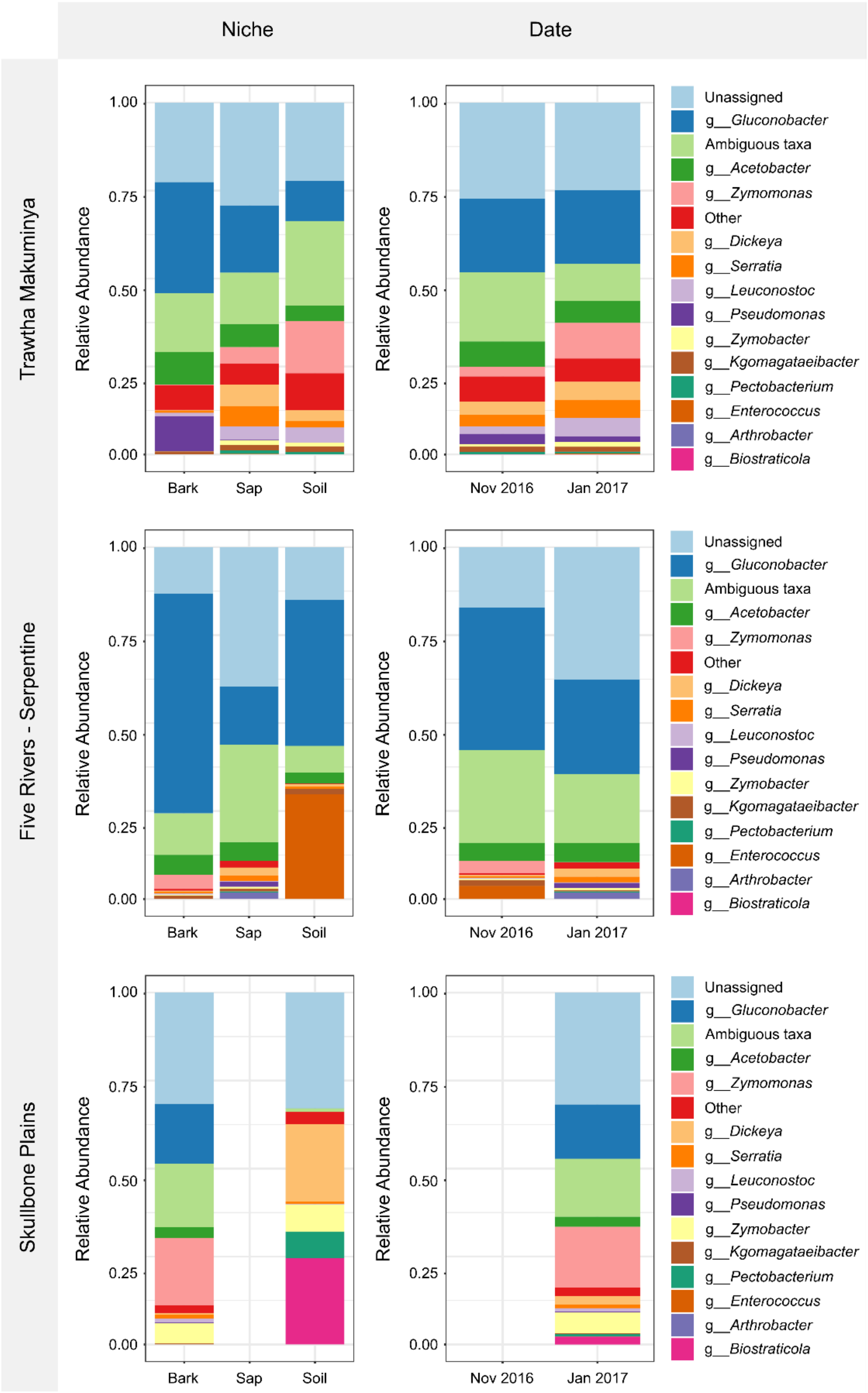

Figure S2. Relative abundance for the most abundant bacterial genera associated with *Eucalyptus gunnii* in Trawtha Makuminya, Skullbone Plains and Five Rivers - Serpentine according to ecological niche, and sampling date.

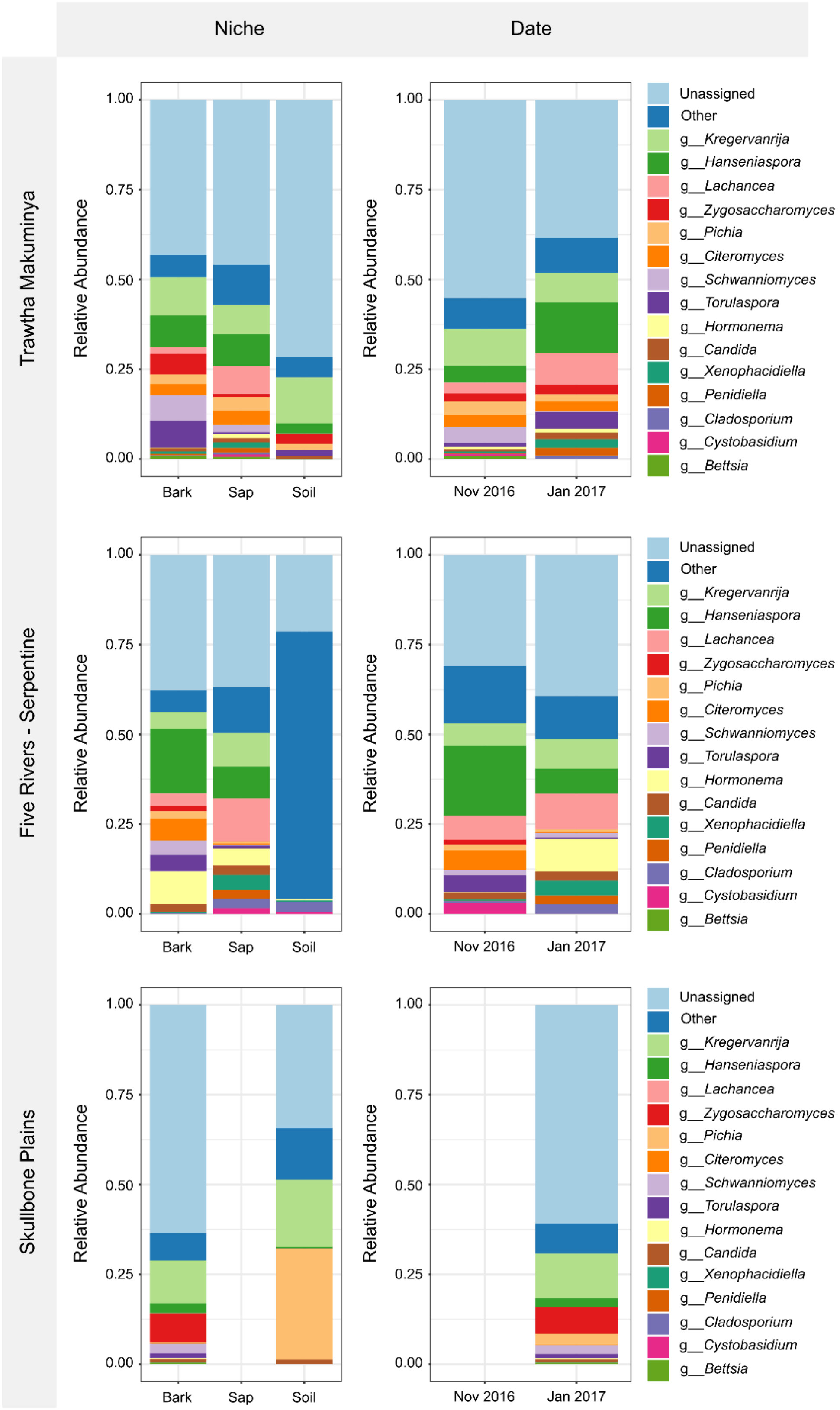

Figure S3. Relative abundance for the 15 most abundant fungal genera associated with *Eucalyptus gunnii* according to geographical location, ecological niche, and sampling date.

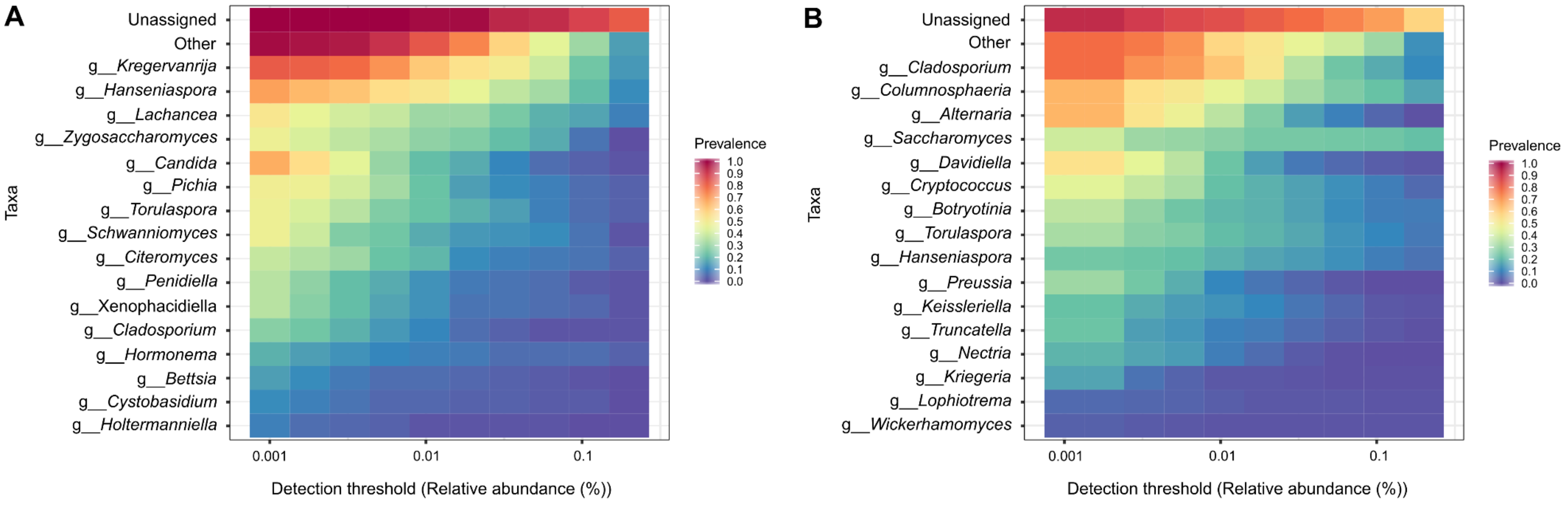

Figure S4. Most prevalent fungal genera associated with *Eucalyptus gunnii* (A) and with *Vitis vinifera* (B). *V. vinifera* data from Morrison-Whittle and Goddard [33,34].

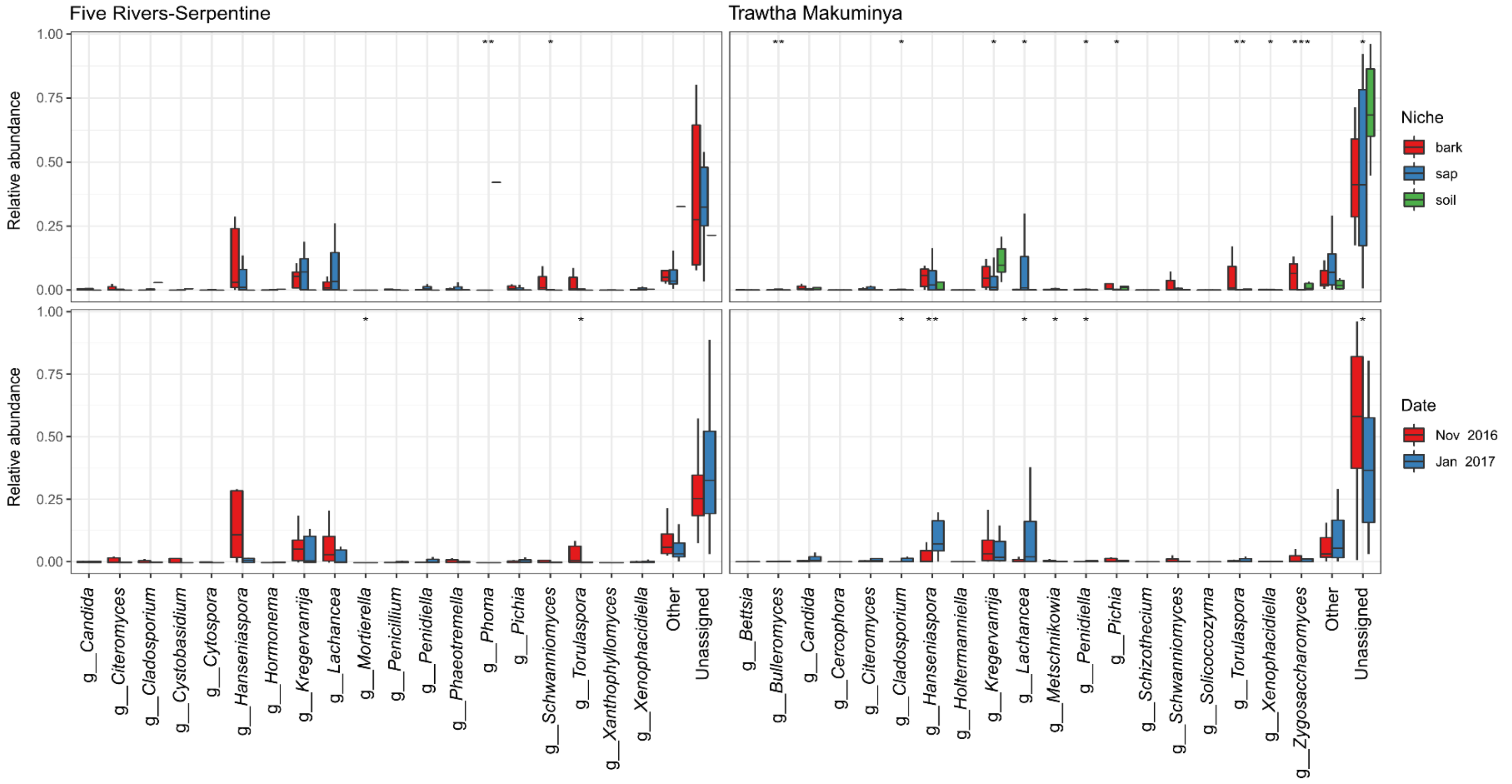

Figure S5. Boxplots showing relative abundance for the 15 most abundant fungal genera in Trawtha Makuminya and Five Rivers - Serpentine according to ecological niche and sampling date. Stars indicate statistically significative differences according to the Kruskal–Wallis test (* $p < 0.05$; ** $p < 0.01$; *** $p < 0.001$).
